# Defined Geldrop Cultures Maintain Neural Precursor Cells

**DOI:** 10.1038/s41598-018-26417-1

**Published:** 2018-05-30

**Authors:** Steffen Vogler, Silvana Prokoph, Uwe Freudenberg, Marcus Binner, Mikhail Tsurkan, Carsten Werner, Gerd Kempermann

**Affiliations:** 1 German Center for Neurodegenerative Diseases (DZNE) Dresden, 01307 Dresden, Germany; 2 CRTD - Center for Regenerative Therapies Dresden, Genomics of Regeneration, Technische Universität Dresden, 01307 Dresden, Germany; 3 Leibniz Institute of Polymer Research Dresden, Max Bergmann Center of Biomaterials Dresden, 01069 Dresden, Germany

**Keywords:** Biomaterials - cells, Neural stem cells

## Abstract

Distinct micro-environmental properties have been reported to be essential for maintenance of neural precursor cells (NPCs) within the adult brain. Due to high complexity and technical limitations, the natural niche can barely be studied systematically *in vivo*. By reconstituting selected environmental properties (adhesiveness, proteolytic degradability, and elasticity) in geldrop cultures, we show that NPCs can be maintained stably at high density over an extended period of time (up to 8 days). In both conventional systems, neurospheres and monolayer cultures, they would expand and (in the case of neurospheres) differentiate rapidly. Further, we report a critical dualism between matrix adhesiveness and degradability. Only if both features are functional NPCs stay proliferative. Lastly, Rho-associated protein kinase was identified as part of a pivotal intracellular signaling cascade controlling cell morphology in response to environmental cues inside geldrop cultures. Our findings demonstrate that simple manipulations of the microenvironment *in vitro* result in an important preservation of stemness features in the cultured precursor cells.

## Introduction

*In vitro* cell culture platforms provide a maximum of reductionism while attempting to mimic key aspects of natural tissue as realistically as possible. Reproducible control over experimental parameters is crucial to dissect a cellular mechanism of interest^1–4^. However, available cell culture systems often do not yet reach that goal.

Adult hippocampal neurogenesis represents an important mechanism of activity-dependent brain plasticity that originates from a somatic precursor cell population that is lifelong maintained in a neurogenic niche in the dentate gyrus. These neural precursor cells (NPCs) can be studied *ex vivo* and two culture forms are in use.

Neurosphere cultures are agglomerate cultures that are easy to grow and maintain but are heterogeneous^5–7^. Monolayer cultures in contrast allow the investigation of relatively homogenous populations^8,9^.

*In vivo*, the lifelong maintenance of NPCs and their responsiveness towards extrinsic stimuli is preserved by a multiparametric regulatory network of niche factors. To have experimental control over cellular and non-cellular regulators is a main goal of *in vitro* platforms. However, the intrinsic reductionism leads to experimental limitations. Specifically, monolayers provide only one-sided and spatially constrained cell-substrate adhesion, which affects downstream, intracellular signaling^10^. Paradoxically, this might lead to signaling that is above or below physiological levels and sets a limit for the maximum number of cells to be cultured. Monolayers are homogenous and highly proliferative, but poor in terms of neuronal differentiation. Neurospheres on the other hand show spontaneous differentiation and are highly heterogeneous^6,11–13^. Both characteristics are disadvantageous for the maintenance of NPCs *in vitro*. In general, both cell culture platforms are not suitable to stably culture NPCs *in vitro* at high densities. Especially the regulatory influence of the extracellular matrix (ECM) is largely neglected, although recent studies have shown the importance of the ECM for NPCs maintenance. Specifically, cell adhesion^14–16^, proteolytic degradability^17,18^, and matrix elasticity^19^ can act as fundamental regulators. Neither monolayers nor neurosphere cultures allow precise control of these factors. Novel cell culture substrates, however, do.

Polymer hydrogels displaying ECM-features such as adhesiveness, proteolytic degradability, and elasticity recommend themselves for deciphering cell-ECM interactions under defined conditions *in vitro*^20–24^. In a remarkable recent example, Caiazzo *et al*.^25^ used a hydrogel-aided cell culture platform to identify matrix-intrinsic factors that increase the reprograming efficiency during generation of induced pluripotent stem cells^25^.

In our study, *in situ* forming covalent polymer networks consisting of 4-arm poly (ethylene glycol), the glycosaminoglycan heparin and functional peptides^26,27^ were used for embedding NPCs in droplet-shaped hydrogel bodies. ECM-features of the hydrogel matrix were systematically varied and adjusted in ways to maximize the maintenance of NPCs.

## Results

### Geldrop Culture in Comparison to Monolayers and Neurospheres

**Figure 1:**
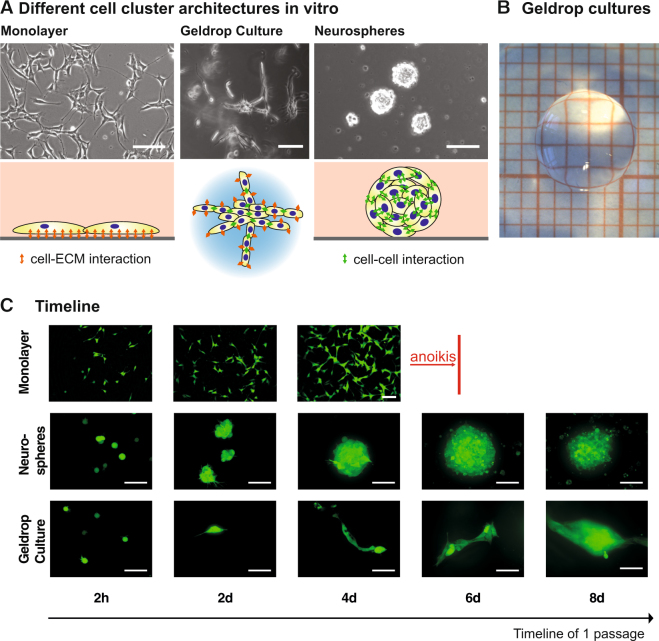
Comparison of conventional with the novel geldrop culture platform. (**A**) Principle cell cluster architecture found in two conventional cell culture platforms (monolayer and neurospheres) and in geldrop cultures with highlighted cell-cell and cell-ECM interactions. Scale bar is 10 µm. (**B**) Brightfield photographic micrograph of a single geldrop as they were used in our experiments. The side length of each square in the background is 1 mm. (**C**) Timeline showing GFP-positive (under β-actin promoter) neural precursor cells in monolayer, neurosphere and geldrop culture. All scale bars are 50 µm.

Appearance of NPC cultures in the commonly applied monolayer and neurosphere versions differs with respect to the arrangement of individual cells (Fig. 1A): Monolayer culture on an adhesive surface enforce elongated cell morphology and result in detachment and anoikis as soon as confluency is reached^28^ Fig. 1C). Neurosphere cultures, in contrast, enable unrestricted proliferation in dense, spherical clusters. However, with increasing size of the neurospheres, concentration gradients of growth factors in the core lead to spontaneous differentiation and eventually apoptosis.

As a third approach, we here introduced a culture type that relies on growing NPCs embedded in small (V = 20 µl) volumes of adhesive, enzymatically cleavable biohybrid hydrogels (Fig. 1A,B). Our resulting “geldrop culture” induced the development of elongated multi-cellular cluster of cells (Fig. 1A,C), enabled growth of cell clusters over an extended period of time and allowed for expansion of NPCs in 3D even at high densities. Direct comparison showed sustained cell cluster growth in the geldrop cultures but not in monolayer and neurosphere cultures over a period of 8 days (Fig. 1C). After day 8, previously separate cell clusters merge and form unified cell agglomerates. At this phase, microscopic analysis becomes impossible, because the endogenous GFP-signal cannot be attributed to individual cells anymore.

**Figure 2:**
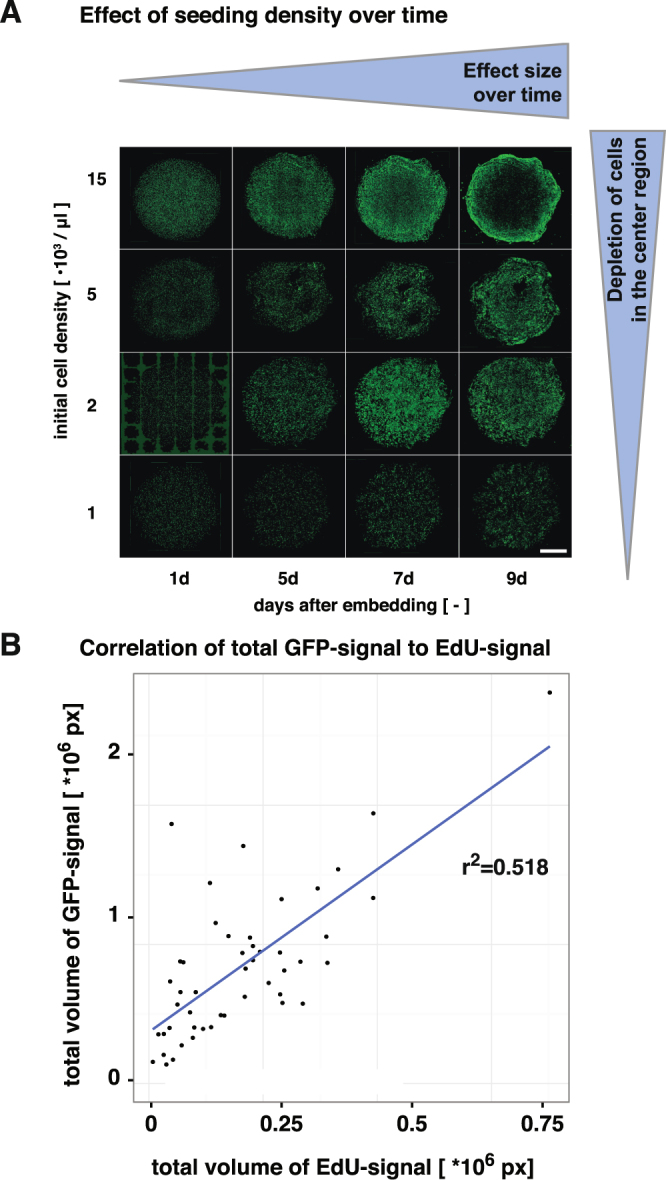
Assessment of Methodological Limitations. (**A**) Effect of seeding density with increasing time in culture (gel volume is constant for all samples). (**B**) Regression analysis shows moderate correlation between total volume of GFP signal and volume of EdU signal (as proxy for cell proliferation). Note that due to absence of cell segmentation, there is no error caused by misidentification of cells.

Pilot studies had revealed that an initial seeding density as low as 1000 cells/µl was sufficient to allow for diffusional growth factor supply even upon sustained cell proliferation (this is confirmed by reports on theoretical estimates of growth factor deficits inside cell-seeded 3D constructs to scale with diffusion distance and density-dependent consumption^29^). Figure 2A depicts the appearance of the gel drop culture as function of cell density and time. The figure suggests that seeding density can be optimized to result in healthy and lasting cultures, in the example a density of 2 would be considered best. Note, however, that these are relative statements, dependent on the assumption of the experiment. Under the set of parameters chosen here, cultures could be stably maintained for approximately 8 days. At this time point the drops started to collapse, resulting in very dense, indistinguishable cell clumps.

### Impact of Hydrogel Characteristics on Geldrop Culture

**Figure 3:**
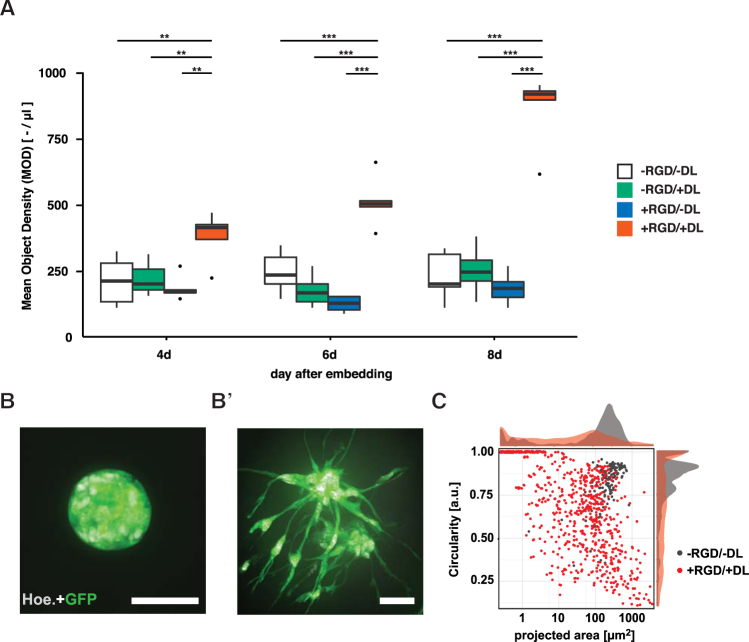
Cellular responses controlled by hydrogel properties. (**A**) Plot representing detected object density for 2 different hydrogel modifications at day 4, day 6, and day 8 (n = 9). Asterisks indicate statistical significance as followed: *(p < 0.05), **(p < 0.01), or ***(p < 0.001). Boxplot shows median and 25th/75th percentile. (**B**) Spherical cluster of NPCs at day 6 after embedding in −RGD/−DL hydrogel. Scale bar is 25 µm. (**B’**) Cluster of NPCs with irregular shape at day 6 after embedding in +RGD/+DL hydrogel. Scale bar is 25 µm. (**C**) Scatterplot depicting circularity and projected area for each detected object imaged in −RGD/−DL and +RGD/+DL hydrogels on day 6 post embedding.

On days 4, 6, and 8 after embedding, the number of detected cell clusters was significantly higher in hydrogels containing an adhesion-promoting peptide and a MMP-degradable linker (i.e.+RGD/+DL) than in any other hydrogel composition (Fig. 3A; for day 4 p < 0.01, for day 6 and 8 p < 0.001, two-way ANOVA F_(6,75)_ = 14.76). A strong pro-proliferative effect was seen only in cultures using gels offering both adhesiveness and degradability. Quantification was conducted on a per object-base, which does not necessarily identify individual cells but instead isolated objects (such as cell clusters for example), so that in terms of cell numbers we might underestimate the absolute values. If one of the hydrogel signals, RGD or DL, was missing (−RGD/+DL or +RGD/−DL), the object density remained on the level of the non-functionalized matrix (−RGD/−DL). The difference in object density between the fully functionalized +RGD/+DL hydrogel and all other gel types became larger over time with an 1.8-fold increase on day 4, an 2.8-fold increase on day 6 and a 3,9-fold increase on day 8 (Fig. 3A). To assess if the object density would indeed be indicative of cell proliferation, an additional experiment was performed (Fig. 2B). The volume of GFP signal (delineating the cell) was plotted over the volume of the (nuclear) EdU signal. Regression analysis revealed an r^2^ = 0.518.

Embedding NPCs in hydrogels without additional functionalization (i.e. −RGD/−DL) resulted in the formation of spherical clusters similar to neurospheres (Fig. 3B). In contrast, in +RGD/+DL hydrogels cell clusters had a branched fine-structure, never seen in the other culture types. All imaged objects in −RGD/−DL hydrogels in a given experiment remained within a small range of size and circularity (Fig. 3C). NPCs grown in +RGD/+DL hydrogels were predominantly found in clusters smaller than in −RGD/−DL hydrogels, and of a higher variability in size. Cell clusters in +RGD/+DL hydrogels were predominantly ovoid to elongated, suggesting maximized interaction with and active colonization of the functionalized hydrogels.

Interestingly, the availability of both adhesion-promoting peptides and enzymatically degradable peptide linkers were critical for cell responses in geldrop cultures even at culture periods of up to 8 days and presumably beyond.

**Figure 4:**
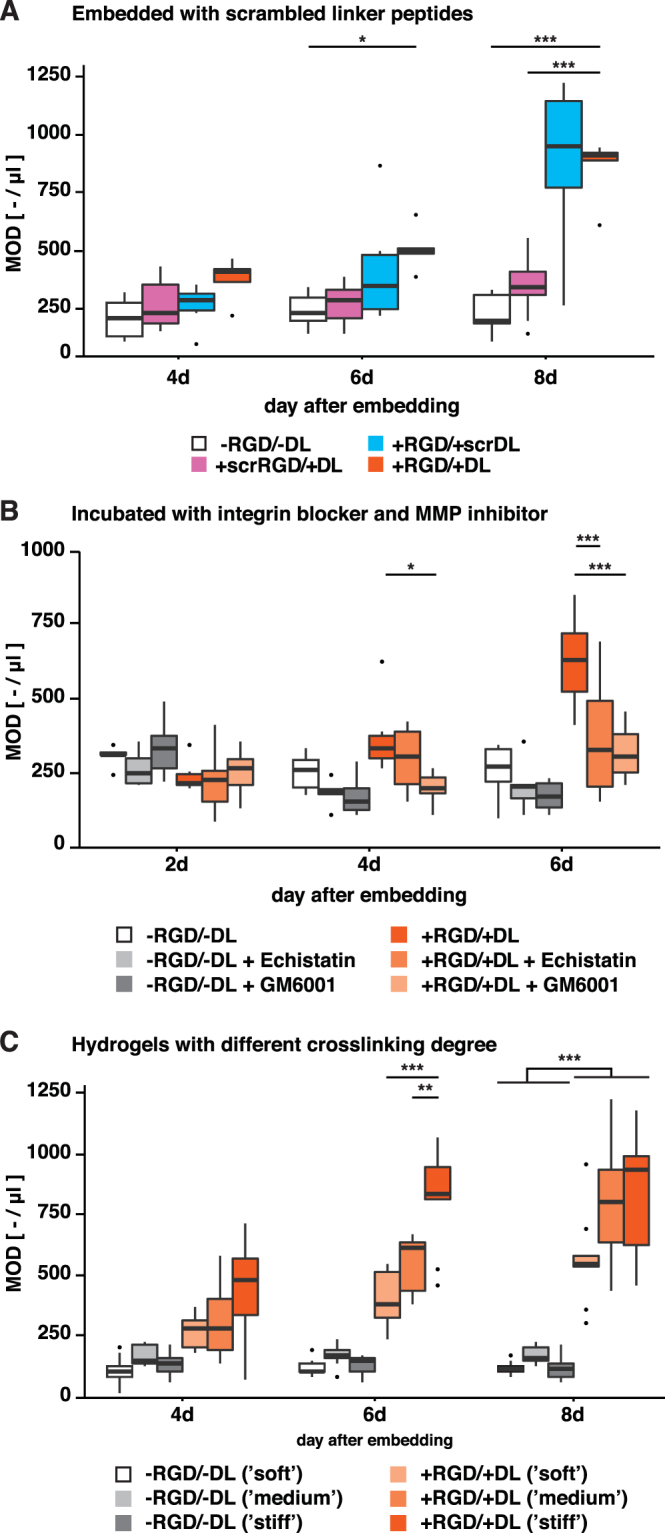
Graded proliferative effect upon manipulation of cell-ECM interaction. (**A**) Graph shows OD for hydrogels with scrambled RGD and scrambled DL plus −RGD/−DL and +RGD/+RGD references. (**B**) OD for hydrogels that were incubated with echistatin (integrin blocker) and GM6001 (MMP-inhibitor) plus −RGD/−DL and +RGD/+DL references. (**C**) OD for hydrogels of different crosslinking degree γ (molar ratio of PEG to heparin-maleimide) for −RGD/−DL and +RGD/+DL hydrogels. n = 9. Asterisks indicate statistical significance as followed: *(p < 0.05), **(p < 0.01), or ***(p < 0.001). Boxplots show median and 25th/75th percentile.

Next, we asked whether manipulations of cell-microenvironment interactions could be used to control the embedded precursor cells. Experiments with scrambled peptides were performed in order to evaluate the specificity of the maintenance effect observed in the +RGD/+DL hydrogels. If scrambled variants of the RGD- and the DL-peptides were used (Fig. 4A), the hydrogel completely lost its pro-proliferative effect (for day 8, OD_+scrRGD/+DL_ = 347.6 ± 42.2 µl^−1^ and OD_−RGD/−DL_ = 226.8 ± 26.6 µl^−1^, two-way ANOVA F_(6,75)_ = 8.23). In contrast, when regular DL was substituted by a scrambled version, the proliferative effect remained unaltered (for day 8, OD_+RGD/+scrDL_ = 883.8 ± 144.4 µl^−1^ and OD_+RGD/+DL_ = 854.2 ± 61.7 µl^−1^).

Next, the specificity of the interactions between the NPCs and the engineered hydrogel matrices was tested. Figure 4B graphically summarizes cellular responses upon addition of an integrin-β1 blocker (Echistatin) and a MMP-inhibitor (GM6001). Both, inhibition of RGD-mediated signaling and MMP-degradation, significantly decreased object density in +RGD/+DL hydrogels measured on day 6 (OD_+RGD/+DL_ = 623.1 ± 64.3 µl^−1^ against OD_+RGD/+DL+Echistatin_ = 363.3 ± 50.5 µl^−1^ and OD_+RGD/+DL+GM6001_ = 317.1 ± 23.8 µl^−1^; both p < 0.001; two-way ANOVA F_(10,126)_ = 6.94) but not in −RGD/−DL geldrop cultures. This confirmed that, in order to be responsive towards the ECM, the NPCs need to be able to (i) receive integrin signals and to (ii) modulate the architecture of their surrounding ECM. The cell capability of the cells to invade the surrounding hydrogel is largely dependent on the activity of MMPs.

Furthermore, we evaluated the effect of hydrogel elasticity on the characteristics of embedded NPCs (Fig. 4C). Hydrogels were used with different degrees of crosslinking (γ 0.75, γ 1.0, and γ 1.5; molar ratio of PEG to heparin-maleimide) and therefore different elasticities (shear moduli G’_γ0.75_ ≈ 0.6–1.6 kPa, G’_γ1.0_ ≈ 1.6–4.0 kPa, and G’_γ1.5_ ≈ 5.8–10.2 kPa) but similar biomolecular functionalization (Tsurkan *et al*.^27^). Cells turned out to be only sensitive to variation in stiffness when cultured in +RGD/+DL hydrogels (Fig. 4C), not the other combinations. Here, at day 6 the highest cell density was found in the stiffest γ1.5 hydrogel (OD_+RGD/+DL_ = 824.7 ± 67.7 µl^−1^). At day 8, the two gel matrices with medium and stiff mechanical properties had equally high cell densities (for γ1.0: OD_+RGD/+MMP_ = 813.5 ± 85.5 µl^−1^, for γ1.5: OD_+RGD/+MMP_ = 824.6 ± 90.0 µl^−1^). Since gels with a higher degree of crosslinking contained a greater number of degradable linkers, the proliferation-enhancing effect could either be caused by increased stiffness or by increased matrix degradability.

### Multipotency and Self-Renewal are Maintained in Geldrop Cultures

**Figure 5:**
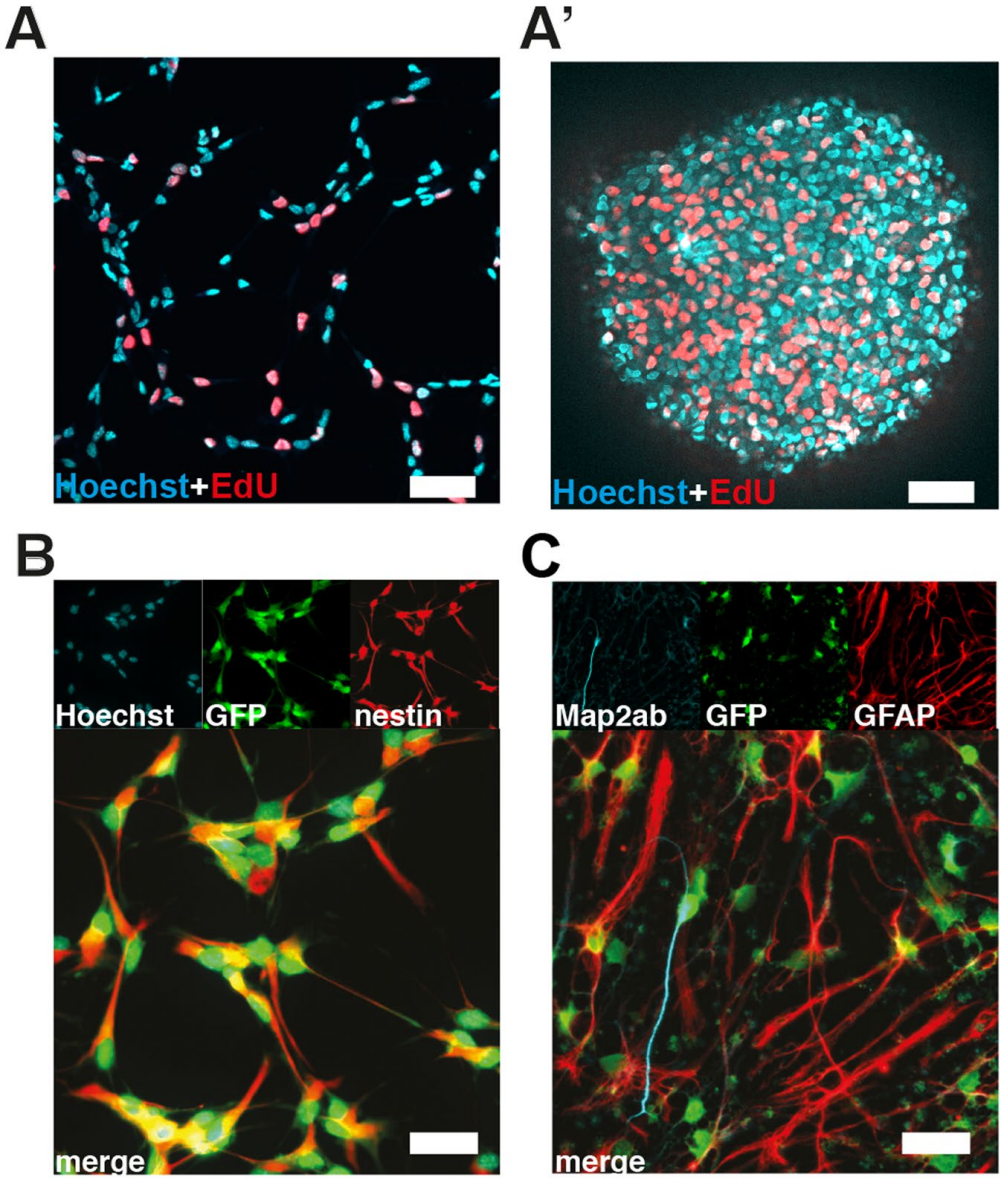
Cardinal features of NPCs are maintained after geldrop culture. (**A**+**A’**) NPCs harvested after 8 days 3D-culture and re-seeded under proliferation condition on a laminin-coated surface (left) and a non-coated surface (right). (**B**) NPCs harvested after 8 days in geldrop culture, re-seeded under proliferation conditions and immunochemically stained for NPC-marker nestin. (**B’**) NPCs harvested after 8 days in geldrop culture, re-seeded under differentiation conditions and immunochemically stained for astrocyte marker GFAP and neuron marker Map2ab. All scale bars are 25 µm.

In order to assess NPC properties after 8 days of geldrop culture, we harvested the NPCs by digesting the gel using Collagenase A and characterized their stemness features by applying standardized proliferation and differentiation assays. Under proliferation conditions and labeling with EdU re-seeded NPCs still showed self-renewal in both conventional monolayer and in neurosphere cultures (Fig. 5A+A’). In addition, all cells were nestin-positive (Fig. 5B). After induction of differentiation by stepwise withdrawal of growth factors^30^, NPCs showed multipotency by differentiating into young neurons (labeled with Map2ab) and astrocytes (labeled with GFAP; Fig. 5C). Assessment of cell differentiation was also attempted by reseeding washed cells (without growth factors) into gel drops, but the incompatibility of the gel-drop with standard immunocytochemistry prevented proper analysis thereafter.

### NPC Behavior in 3D is Regulated by Rho-associated protein kinase (ROCK)

Finally we wanted to find evidence that the observed morphology represents a regulated cellular behavior. Rho GTPases are critical in controlling cell morphology and migratory behavior in many cell types. Rho-associated protein kinase (ROCK) is a kinase and downstream effector of Rho.

**Figure 6:**
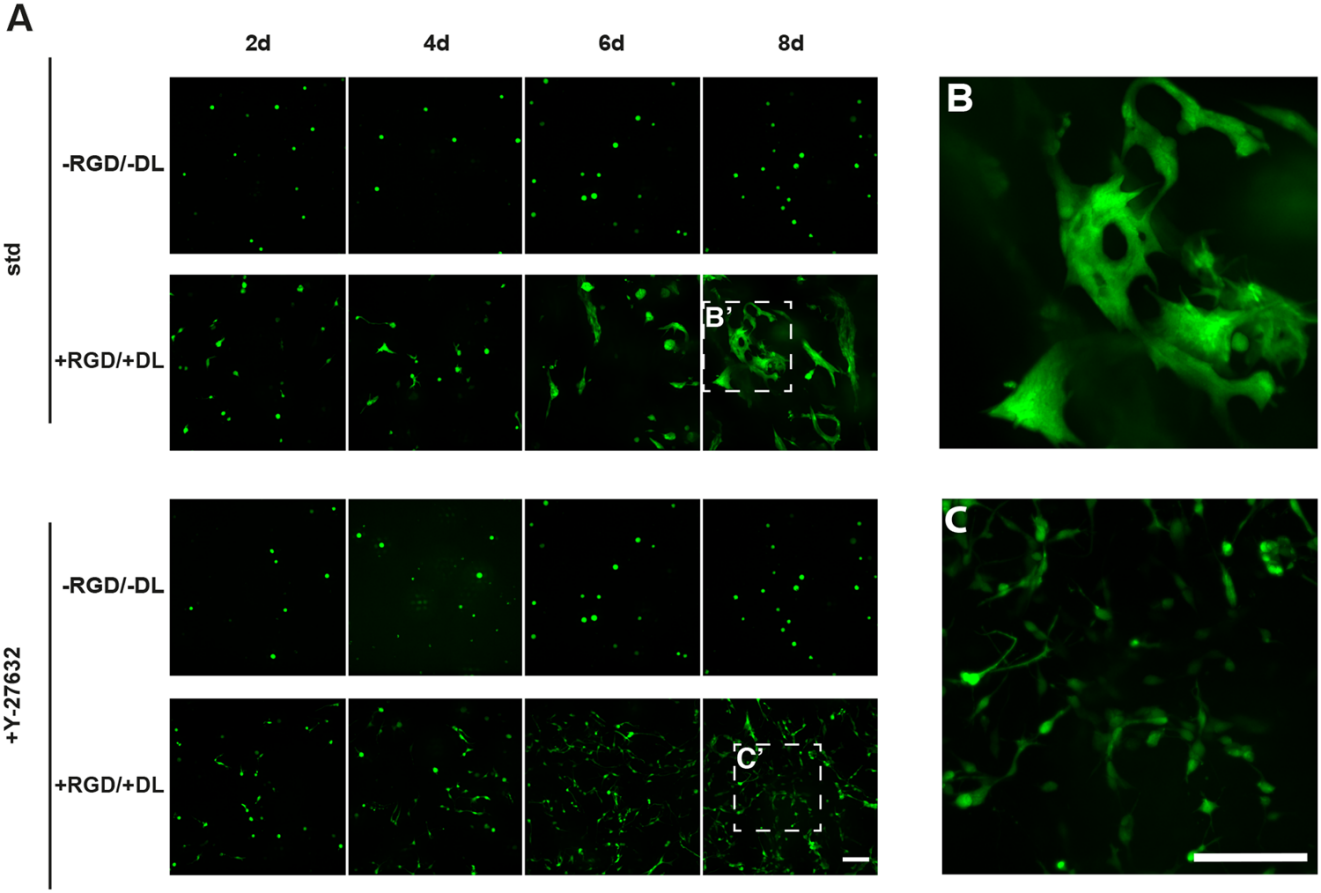
Identification of ROCK as an intracellular regulator of cell morphology. (**A**) Panel shows NPCs in differently functionalized hydrogels (−RGD/−DL and +RGD/+DL) for multiple days in culture under absence (top) and presence (bottom) of ROCK-inhibitor Y-27632. (**B**) NPCs arranged in elongated, irregular-shaped clusters at the end of a culture period in +RGD/+DL hydrogel. (**C**) Neural precursor cells distributed separately with elongated cell morphology at the end of a culture period in +RGD/+DL hydrogel with Y-27632 at 10 µM. All scale bars are 25 µm.

When cultured in −RGD/−DL hydrogels, application of ROCK inhibitor Y-27632 to NPCs showed no response different from controls (Fig. 6A). On the other hand, if NPCs had been embedded in +RGD/+DL hydrogels, morphological differences were detectable in the presence of ROCK inhibitor. The cells showed a dispersed distribution throughout the hydrogel and avoided cell-cell contact, rather than showed the described island- or streak-like patterns of densely packed multi-cellular complexes (Fig. 6B). In addition, ROCK inhibition induced an elongated, polarized morphology, which might be indicative of changes at the cytoskeletal level (Fig. 6C). The fact, that an effect of ROCK inhibition was only visible in +RGD/+DL hydrogels, strongly suggests that ROCK acts in concert with parts of an integrin-mediated signaling pathway.

## Discussion

Our geldrop culture experiments show that adding extracellular cues profoundly alters how NPCs act in culture. Most importantly, the novel culture environment allows to maintain them in a stem-like state with low rates of baseline expansion in which the cells, other than commonly observed under neurosphere conditions, do not show signs of instantaneous differentiation. Monolayer cultures do not show spontaneous differentiation as long as they are kept in the presence of growth factors.

Culturing NPCs in geldrops also resulted in a distinct streak-like cellular arrangement. The cells with their characteristic bipolar shape, similar to the ones seen under monolayer conditions, were often aligned in parallel, forming bands of cells. This pattern is impossible to induce in conventional cell culture platforms and is reminiscent of migratory cells during development, e.g. of the dentate gyrus (compare^31,32^). The cells show an elongated morphology, resembling the picture in monolayer cultures, but are often aligned in parallel and actual migration will likely be limited due to the mechanical properties of the drop and the dense cell mass.

Specifically, the densely packed and stretched-out cell clusters combined the two hallmarks of monolayer (only cell-ECM interaction) and of neurospheres (only cell-cell interaction) in a characteristic, previously undescribed manner. We found that this arrangement stabilized the cell population, which did not collapse upon sustained proliferation as usually seen in conventional cell culture platforms. In monolayer cultures, NPCs proliferate until confluency is reached, then cells collectively detach from the surface and undergo apoptosis (anoikis)^28^. Thus, an adhesive 2D substrate appears to be a factor that both promotes and limits proliferation. These conditions set an upper limit in terms of final cell density as well as for the possible time in culture without passaging. In contrast, neurospheres do not rely on the availability of adhesive substrates and are consequently not restricted in this respect. Nevertheless, the naturally resulting spherical shape of the cell clusters is not beneficial for the continued supply by growth factors by diffusion. More precisely, as the NPCs maintain proliferative, the volume of the sphere increases. From a purely geometrical point of view, doubling the diameter of the sphere increases the surface by a factor of 4, whereas the volume increased by a factor of 8. As a consequence, growth factor supply through the surface becomes increasingly insufficient with greater spheres. Hence, in analogy to the monolayers, the proliferation of the NPCs eventually reaches an upper limit of cell numbers that can be maintained. However, arrangement of NPCs in elongated clusters as in the geldrop cultures presumably assured a beneficial surface-to-volume ratio.

Methodological attention needs to be paid to the metric of ‘object density’ used in Fig. 3 and elsewhere. Because of high local cell densities and the fact that the GFP signal delineates the entire soma, it is not possible to estimate the exact number of cells per cluster. Instead, each spatially separated cluster has been counted as a single object. In order to assess the extent of error, the ‘GFP-to-EdU relationship’ has been estimated to test, to which extend cell cluster size is predictive to assess cell proliferation (Fig. 2B). The comparison is based on two volumetric measures, which in the case of the nuclear EdU-signal is an unusual approach because the volume does not directly translate into a count. This would require object segmentation, which is highly error-prone for densely packed cells. But under the assumption that the volume of a nucleus is constant, the total amount of EdU-volume is a linearly-dependent measure for proliferating cells without the error through mis-segmentation. The fit of a linear model allows the conclusion that measuring GFP-signal is a valid proxy for proliferation.

Size of the drop is an important parameter. The bigger the drop, the longer is the “diffusion distance” and the less sufficient is the supply of NPCs residing at the center with growth factors. Thus, NPCs can be maintained longer, if they are seeded at a lower concentration (compare Fig. 2A), whereas the total gel volume must not exceed 25 µl. These observations roughly match a theoretical estimate reported by McMurtrey^29^.

A disadvantage of the gel drop cultures is that immunocytochemistry is often impossible, presumably due to antibody scavenging and penetration issues. This limits the use of markers that can be applied. Staining for cytoskeletal markers, for example, was not successful, so that our conclusions about the nature of cytoskeletal changes, also in response to ROCK inhibition remains limited.

By control of cell cluster architecture, sustained proliferation of cells no longer leads to rapid (in monolayer) or subtle (in neurospheres) collapse of the NPC population. Instead it is possible to steer cell cluster growth towards a state that allows sufficient presentation of adhesion sites and diffusible growth factors.

Further, we here show that presentation of adhesion ligands and susceptibility to enzymatic attack of the hydrogel matrix (i.e +RGD/+DL hydrogels) are essential for sustained proliferation of NPCs. If either one of the two cues or both were absent, the cells are were no longer able to arrange in the elongated clusters that sustained proliferation. This finding highlights a fundamental dualism of matrix adhesiveness and degradability. In the case of −RGD/−DL hydrogels, cell cluster architecture was similar to that of neurospheres. There, cells proliferate in absence of adhesion sites and degradable peptides and cell cluster size increases purely due to undirected expansive growth^33^. Hydrogels presenting only one of the functionalization motifs (i.e. either RGD or DL) gave rise to results similar to those of non-functionalized hydrogels. Of note, Ehrbar *et al*. similarly showed that effective migration of cells in hydrogel depended on adhesive cues and degradability^34,35^. This is further supported by other reports that cell adhesion^14–16^ and MMP-mediated degradation^17,18^ are potent regulators of NPC activity.

Scrambling the RGD-sequence, which prevents integrin-specific cell adhesion, erased the low but distinctive pro-proliferative effect of the hydrogel, indicating that functional cell-ECM binding is crucial for NPCs to receive instructive cues from their microenvironment. This idea is supported by reports by Shen *et al*.^15^ and Loulier *et al*.^16^, which showed that the positive effects of cell adhesion on NPC proliferation are integrin-specific. Importantly, however, scrambled degradable linkers did not lead to a decrease in proliferation. It remains to be seen if NPCs can indeed degrade surrounding hydrogel insensitive to amino-acid sequence as such data might suggest, whereas cell adhesion is a sequence-sensitive mechanism. In a different setting, an outside-in integrin-signaling was required for exocytosis of MMP-2 by human melanoma cells^36^. In addition, Gupton *et al*. have shown the importance of integrin-controlled exocytosis during neuritogenesis in adherent neural cells^37^. Generally this suggests an inherent hierarchy among environmental cues, with integrin-signaling being the basis for exocytosis events. This could explain why NPCs did not respond differentially between −RGD/−DL and −RGD/+DL hydrogels (see Fig. 4C).

In addition to the instructive cues from the surrounding hydrogel matrix, we investigated to which extent cell-intrinsic features influenced cell-ECM interaction. Specifically, we blocked recognition of matrix-bound RGD via addition of echistatin (a potent inhibitor of integrin β1 and integrin β3) and saw a decrease in cluster density. In analogy, proteolytic degradation has been specifically blocked through the addition of GM6001 – a small molecule inhibitor affecting a wide subset of MMPs. Once MMP activity was decreased, the measured object density dropped – although the hydrogel was functionalized with both RGD and DL. This observation allows the conclusions, that NPCs cleave the surrounding matrix mainly using MMPs. However, as seen in the experiment with scrambled DL (Fig. 4A), this proteolysis seemed robust towards changes in the sequence of the substrate.

In summary, maintenance of NPCs in +RGD/+DL hydrogels relied on a functional cell-microenvironment interaction. We observed a bi-directionality of this interaction: perturbation on both the matrix side (by scrambled linker peptides) and on the cell side (by molecular inhibitors) equally led to a loss of the maintenance effect.

In order to validate one of the potentially involved signaling pathways in the intracellular transmission of incoming environmental cues, we tested ROCK as important candidate. Inhibition of ROCK did not have an effect when NPCs were cultured in −RGD/−DL hydrogels, but as soon as NPCs were cultured in +RGD/+DL hydrogels the role of ROCK in cell-cell adhesion and the regulation of cell morphology became apparent. With functional ROCK, NPCs were in close contact to neighboring cells and tended to arrange in elongated clusters with an obvious directional preference. Once ROCK was inhibited by Y-27632, NPCs did not arrange in densely packed cell cluster and tended to project into the matrix while possessing an elongated cell shape, which is in line with findings by others^38^. The decrease in cell-cell interaction might be explainable by a decrease in cadherin protein upon ROCK inhibition^39^.

We have used secondary cultures to assess potential downstream consequences of geldrop culturing. However, we saw a complete maintenance of the cardinal stem cell properties. Neither multipotency nor self-renewal potential were lost (+RGD/+DL hydrogel was used). This does not imply that gel drops would leave the cells completely unaltered, but unlike in the monolayer or neurosphere situation their stemness activity was not exhausted during the experimental time period. Yang *et al*. for example showed that at a proteomic level changes in response to a distinct mechanical environment were preserved long-term^40^. This cellular ‘mechanical memory’ influenced cell fate decision at a later time point, even within a different environment. In the context of cultured NPCs there is thus a need for additional studies targeting the proteomic and transcriptomic level to further clarify, if and how the cells are intrinsically affected by their culture microenvironment. Generally, results from secondary cultures highlight the potential of the geldrop culture to strongly instruct cells during the period of culture while in parallel maintaining their cardinal features – a fundamental requirement for the *in vitro* maintenance of NPCs.

In summary we have demonstrated the applicability of geldrop cultures to maintain NPCs at high density *in vitro*. The specific advantage of that approach over monolayer and neurosphere cultures originates from an *in vivo*-like representation of the microenvironment. In this arrangement, cell-cell and cell-ECM interactions can act in concert rather than excluding each other (as seen in the two extreme conventional platforms). It was possible to systematically vary matrix-dependent signals and thereby optimize the microenvironment for NPCs. An optimized hydrogel matrix then allowed cell cultivation under high cell densities in a chemically defined 3D environment without losing the multipotency and self-renewal potential. While the incompatibility of the hydrogel with immunohistochemistry poses a serious practical limitation, the fact that precursor cells can become stabilized in a reductionistic artificial microenvironment is an important step towards building synthetic stem cell niches.

The stabilization and localized presentation of growth factors by the highly sulfated glycosaminoglycan heparin used as a building block of the hydrogel network can be furthermore assumed to contribute to the beneficial effects of the 3D geldrop culture^41–43^. Variation of the glycosaminoglycan component^44^ as well as customized pre-loading of the gels with defined combinations of growth factors^45^ can therefore be considered powerful additional options of the system clearly exceeding the possibilities of the conventional culture schemes.

## Methods

### Adherent Cell Culture

Transgenic Actin-EGFP mice (6-weeks-old; 6 animals per isolation; ref.^46^) were used for isolation of hippocampal neural precursor cells (NPCs). Mice were killed with an overdose of anesthetics, the brains were collected and the dentate gyri were microdissected^30,47^. Using the Neural Dissociation Kit (Miltenyi Biotec) the tissue was enzymatically digested. The procedure was conducted according to the manufacturer’s instructions. The resulting cell pellet was resuspended in 0.5 ml Neurobasal medium (NB, Gibco) with B27 supplement (Gibco), 20 ng/ml EGF and 20 ng/ml bFGF (both from Peprotec). Then the cell suspension was transferred into a poly-D-lysine and laminin-coated well of a 24-well tissue culture plate. NPCs cells were cultured in humidified 5% CO_2_ at 37 °C. Medium was changed every 2 days. When cells reached confluency of 80% they were passaged by incubation with accutase for 3 min, subsequent centrifugation and reseeding into the next culture vessel. When reaching passage 5, the cell population could be routinely considered to contain a pure NPC population (as based on Nestin/Sox2 expression, ref.^30^).

The experiments were conducted in accordance with the applicable European and National regulations (Tierschutzgesetz) and approved by the responsible authority (Regierungspräsidium Dresden).

### Geldrop Cultures

Droplet-shaped hydrogel bodies were formed by click reaction chemistry of peptide-conjugated, thiol-functionalized 4-arm polyethylene glycol (starPEG) and maleimide-functionalized heparin as described elsewhere^27^. A heparin batch with an average of 6 maleimide groups per molecule heparin was used for all experiments. The crosslinking degree γ (molar ratio of PEG to heparin-maleimide) of the polymer network was adjusted to modify the mechanical properties of the hydrogel. Specifically, a ‘soft’ hydrogel was achieved with crosslinking degree γ = 0.75-‘medium’ is γ = 1.0 and ‘stiff’ is γ = 1.5. Adhesiveness of the hydrogel was controlled by conjugating heparin with adhesion ligand protein-derived peptides. Proteolytic degradability was achieved by terminal functionalization of 4-arm PEG with MMP-cleavable peptide substrates^27^. For embedding of NPCs, a cell suspension was prepared as described above and kept on ice until further processing. Adhesion peptide sequences were immobilized on a previously prepared solution of heparin-maleimide conjugate (in PBS) by adding reactive peptide in a ratio of 2 mol peptide to 1 mol heparin-maleimide solution. After vortexing, the mix was kept continuously on ice. Then 4-arm PEG was dissolved in PBS and within 3 min after preparation of a mix of cell suspension and heparin-maleimide, a gel drop was formed by mixing the cell-heparin-mix with equal volume of the 4-arm PEG precursor solution. After 5 times repeated pipette aspiration, a 20 μl drop was transferred onto the bottom of a 24-glass bottom well plate, where gelation completed. Finally, the cell-containing gel drop was submerged in proliferation medium followed by a complete media exchange after 1 hour. Samples were kept under standard culture conditions. Half-medium changes containing doubled growth factor concentrations were performed every second day. Table 1 lists the hydrogel components used in this project. For the following discussion “RGD” refers to the adhesion sequence and “DL” refers to the degradable. Presence of functional groups within a respective hydrogel is indicated by +/− prefix in the text. For inhibition of Rho-associated protein kinase (ROCK) the inhibitor Y-27632 (Sigma-Aldrich) was added at a concentration of 10 µM.

**Table 1:** Hydrogel components.

Name	Terminal peptide sequence	Function
HM	—	maleimide-functionalized heparin (molar ratio: six maleimides per heparin)
PEG-SH	—	non-degradable backbone
PEG-DL	GCGGPQGIWGQGGCG	PEG w/degradable linker
PEG-scrDL	GCGIGQGQGPWGGCG	PEG w/modified degradable linker
RGD	GCWGGRGDSP	adhesion site
scrRGD	GCWGGRADSP	modified adhesion site

### Gel Digestion and Secondary Cultures

First, Collagenase A was dissolved at 1 mg/ml in complete Neurobasal medium. Then gels possessing a MMP-cleavable peptide sequence were digested by incubation with Collagenase solution for 5 min at 37 °C. Remaining cell aggregates were minced by repetitive up-and-down pipetting. Finally, cells were washed in Neurobasal medium, centrifuged (300 × g, 5 min) and seeded at a density of 10^4^ cells/cm^2^. Secondary culture was characterized by immunochemical staining upon proliferation and differentiation assay.

### Immunocytochemical Staining

After complete removal of the medium cells were rinsed with PBS. Next, cells were fixed with 4% PFA for 20 min. at room temperature followed by a wash in PBS. Afterwards the samples were permeabilized with 0.1% Triton-X100 (Sigma-Aldrich) for 15 min, blocked with 10% donkey serum for 1 h and incubated with primary antibodies in 3% donkey serum over night at 4 °C. The following primary antibodies were used: mouse anti-Nestin (1:500 dilution, 611658, BD), chicken anti-GFAP (1:500 dilution, ab50738, Abcam), and mouse anti-Map2ab (1:500 dilution, M1406, Sigma-Aldrich). Then samples were rinsed three times with PBS and incubated with secondary antibody solution for 4 h at room temperature. Following secondary antibodies were used: anti-mouse Alexa Fluor 568 (1:1000, Invitrogen) and anti-chicken Cy5 (1:1000, Dianova). Nuclear counter stain was performed via incubation with Hoechst 33342 (1:4000, Sigma-Aldrich) for 15 min. After a final washing step, the samples were stored at 4 °C until image acquisition.

### EdU labeling

Samples were incubated with EdU (5-ethynyl-2′-deoxyuridine) at 2.25 µg/ml in regular medium for 2 hours. Upon fixing with 4% PFA for 20 min at room temperature samples were washed once with PBS. For detection of EdU, a commercial Click-iT EdU imaging kit was used (Cat-#: C10337, Invitrogen). According to the kit’s protocol the samples were incubated for 30 min with the Click-iT reaction cocktail. After one washing step the samples were additionally counterstained with Hoechst 33342 (1:4000) for 15 min and washed 3 times.

### Image Acquisition and Quantification

Live bActin-GFP +-NPCs embedded in hydrogel were imaged under cell culture conditions (37 °C; humidified 5% CO_2_) using a spinning disk microscope equipped with an incubation encasement (Carl Zeiss Microscopy). Fluorescent excitation and image acquisition parameters were balanced for minimum energy input and fast image acquisition. Fluorescence images of fixed cells were acquired using the same kind of microscope without incubation periphery. Acquired z-stacks were of identical size, sampled from random locations inside the gel whilst avoiding the gel-medium interface and the underlying glass substrate. All processing steps applied are included in the open-source software FIJI^48^. Raw images were pre-processed using ‘Background Subtraction’ followed by ‘Gamma Correction’. Next, images were segmented using automated OTSU thresholding. The resulting binary images were than quantified for object counts using the plug-in ‘3D Object Counter’ with a lower size cut-off of 100 voxel (voxel size: d_x_ = 0.645 µm, d_y_ = 0.645 µm, d_z_ = 3.13 µm). Then, the sum of all object counts (derived from multiple z-stacks per geldrop) was divided by the total scanned gel volume that had been investigated by spinning disk microscopy in that experiment. The scanned gel volume can be calculated as area of field-of-view times the height of a z-stack. By normalizing object counts on a per-volume-base, the metric of object density is calculated. Quantification of circularity and area was performed on z-projections of previously masked images (i.e. results of the 3D Object Counter).

### Statistical Analysis

All statistics were performed using KNIME (version 2.10.3) and R (version 3.1.0). Significance levels were assessed using a two-way analysis of variance (ANOVA) using Tukey’s least significant difference as a post hoc test, where appropriate. Data presented in figures shows mean ± standard error of mean (SEM). Asterisks indicate conventional statistical significance as followed: *(p < 0.05), **(p < 0.01), or ***(p < 0.001).
