# Author Correction: Defined Geldrop Cultures Maintain Neural Precursor Cells

**DOI:** 10.1038/s41598-020-63854-3

**Published:** 2020-04-17

**Authors:** Steffen Vogler, Silvana Prokoph, Uwe Freudenberg, Marcus Binner, Mikhail Tsurkan, Carsten Werner, Gerd Kempermann

**Affiliations:** 1 German Center for Neurodegenerative Diseases (DZNE) Dresden, 01307 Dresden, Germany; 2 CRTD - Center for Regenerative Therapies Dresden, Genomics of Regeneration, Technische Universität Dresden, 01307 Dresden, Germany; 3 Leibniz Institute of Polymer Research Dresden, Max Bergmann Center of Biomaterials Dresden, 01069 Dresden, Germany

Correction to: *Scientific Reports* 10.1038/s41598-018-26417-1, published online 30 May 2018

This Article contains an error in Figure 6A where panels 6d and 8d for std, –RGD/-DL are duplicate of panel 6d and 8d with +Y-27632, -RGD/-DL.r. The correct Figure 6 appears below as Figure 1.

**Figure 1:**
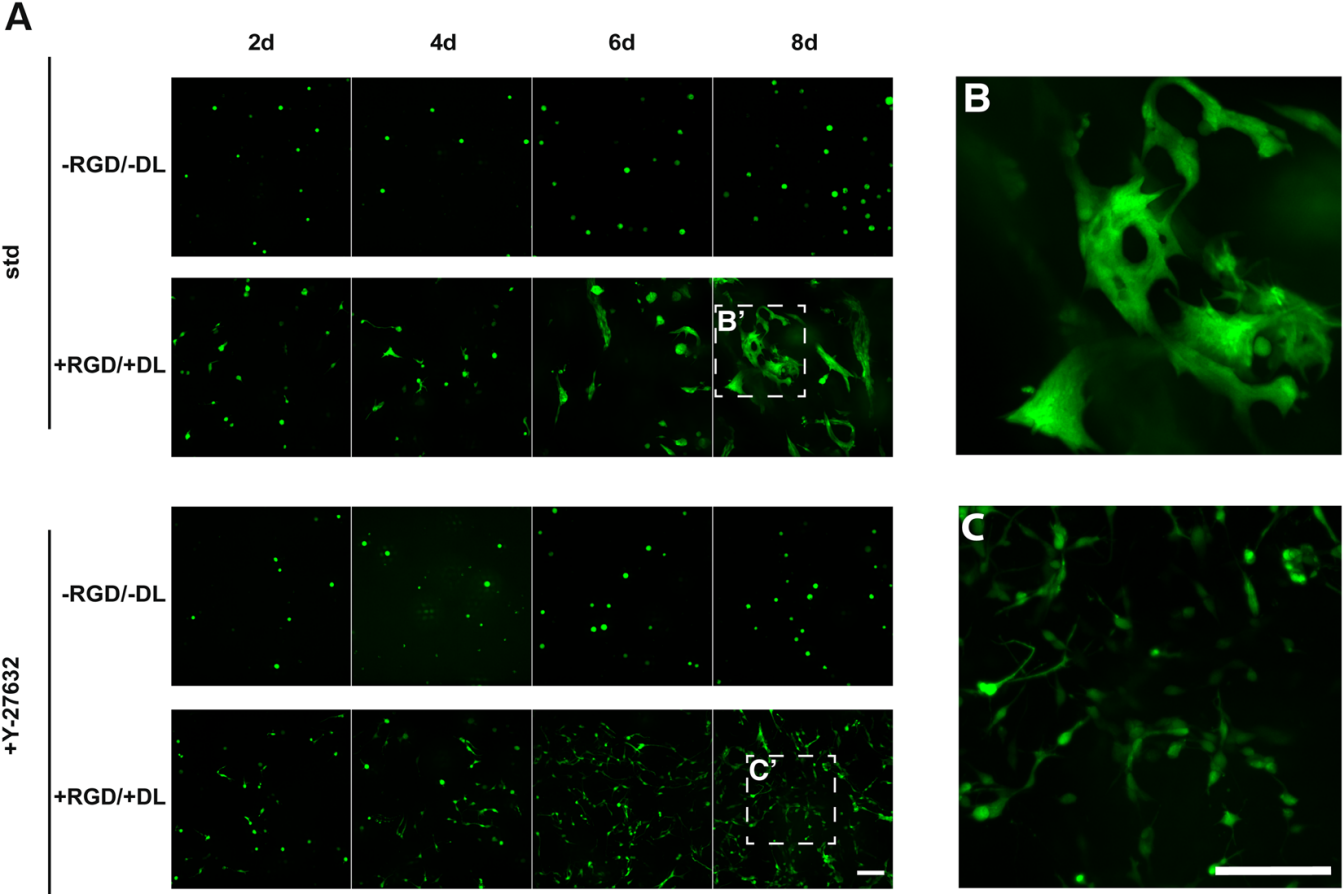
.

